# Prostate Cancer Tumour Features on Template Prostate-mapping Biopsies: Implications for Focal Therapy^☆^

**DOI:** 10.1016/j.eururo.2013.09.045

**Published:** 2014-07

**Authors:** Paras B. Singh, Chukwuemeka Anele, Emma Dalton, Omar Barbouti, Daniel Stevens, Pratik Gurung, Manit Arya, Charles Jameson, Alex Freeman, Mark Emberton, Hashim U. Ahmed

**Affiliations:** a Division of Surgery and Interventional Sciences, University College London, London, UK; b Medical School, University College London, London, UK; c Nuffield Department of Surgical Sciences, University of Oxford, Oxford, UK; d Department of Urology, University College London Hospitals NHS Foundation Trust, London, UK; e Barts Cancer Institute, Queen Mary, University of London, London, UK; f Department of Histopathology, University College London Hospitals NHS Foundation Trust, London, UK

**Keywords:** Prostate cancer, Biopsy, Diagnosis, Pathology, Surgery, Therapy

## Abstract

**Background:**

Focal therapy is being offered as a viable alternative for men with localised prostate cancer (PCa), but it is unclear which men may be suitable.

**Objective:**

To determine the proportion of men with localised PCa who are potentially suitable for focal therapy.

**Design, setting, and participants:**

Our institutional transperineal template prostate-mapping (TTPM) biopsy registry of 377 men from 2006 to 2010 identified 291 consecutive men with no prior treatment.

**Intervention:**

TTPM biopsies using a 5-mm sampling frame.

**Outcome measurements and statistical analysis:**

Suitability for focal therapy required the cancer to be (1) unifocal, (2) unilateral, (3) bilateral/bifocal with at least one neurovascular bundle avoided, or (4) bilateral/multifocal with one dominant index lesion and secondary lesions with Gleason ≤3 + 3 and cancer core involvement ≤3 mm. Binary logistic regression modelling was used to determine variables predictive for focal therapy suitability.

**Results and limitations:**

The median age was 61 yr, and the median prostate-specific antigen was 6.8 ng/ml. The median total was 29 cores, with a median of 8 positive cores. Of 239 of 291 men with cancer, 29% (70 men), 60% (144 men), and 8% (20 men) had low-, intermediate-, and high-risk PCa, respectively. Ninety-two percent (220 men) were suitable for one form of focal therapy: hemiablation (22%, 53 men), unifocal ablation (31%, 73 men), bilateral/bifocal ablation (14%, 33 men), and index lesion ablation (26%, 61 men). Binary logistic regression modelling incorporating transrectal biopsy parameters showed no statistically significant predictive variable. When incorporating TTPM parameters, only T stage was a significant negative predictor for suitability (*p* = 0.001) (odds ratio: 0.001 [95% confidence interval, 0.000–0.048]). Limitations of the study include potential selection bias caused by tertiary referral practise and lack of long-term results on focal therapy efficacy.

**Conclusions:**

Focal therapy requires an accurate tool to localise individual cancer lesions. When such a test, TTPM biopsy, was applied to men with low- and intermediate-risk PCa, most of the men were suitable for a tissue preservation strategy.

## Take Home Message

When transperineal template prostate-mapping biopsy was applied to men with low- and intermediate-risk prostate cancer, most were found to be suitable for a focal therapy, including the strategy of index lesion ablation in multifocal disease.

## 1 Introduction

Localised prostate cancer (PCa) treatment currently involves surgery or radiotherapy applied to the whole prostate regardless of the location or volume of individual PCa lesions. Although there is a survival benefit from this approach in men with intermediate- and high-risk disease, radical whole-gland therapies are associated with a significant risk of rectal complications, incontinence, and impotence [1,2]. Tissue-preserving focal therapy, in which only areas of known cancer are targeted, may improve the therapeutic ratio [3–7]. A number of early-phase studies have shown that preservation of genitourinary function can be high following focal therapy, although cancer control in the medium and long term is yet to be fully evaluated [8–11].

One of the key challenges with focal therapy is to accurately identify the population of men who are potentially suitable for tissue preservation. Some practitioners have argued that focal therapy is an alternative in men suitable for active surveillance [3,5,12], while others have argued that focal therapy should be investigated as a potential alternative to radical therapy in those men likely to benefit from treatment [4,6,12,13]. This argument incorporates the concept of ablating the index cancer lesion, which usually harbours the highest grade and largest cancer volume [14]. A number of ethics committee–approved trials are currently recruiting men with intermediate- and high-risk disease and treating them in an index lesion–ablative manner [15–17].

Therefore, the population of men who are potentially eligible for focal therapy is likely to vary with respect to risk group and is dependent on the focal therapy strategy. Studies using whole-mount prostatectomy specimens to estimate this population might incorporate selection bias, since men would have chosen surgery rather than any number of other treatment modalities. We sought to evaluate the proportion of men suitable for focal therapy based on transperineal template prostate-mapping (TTPM) biopsies, as this test can be applied to all men prior to treatment.

## 2 Methods

**Fig. 1:**
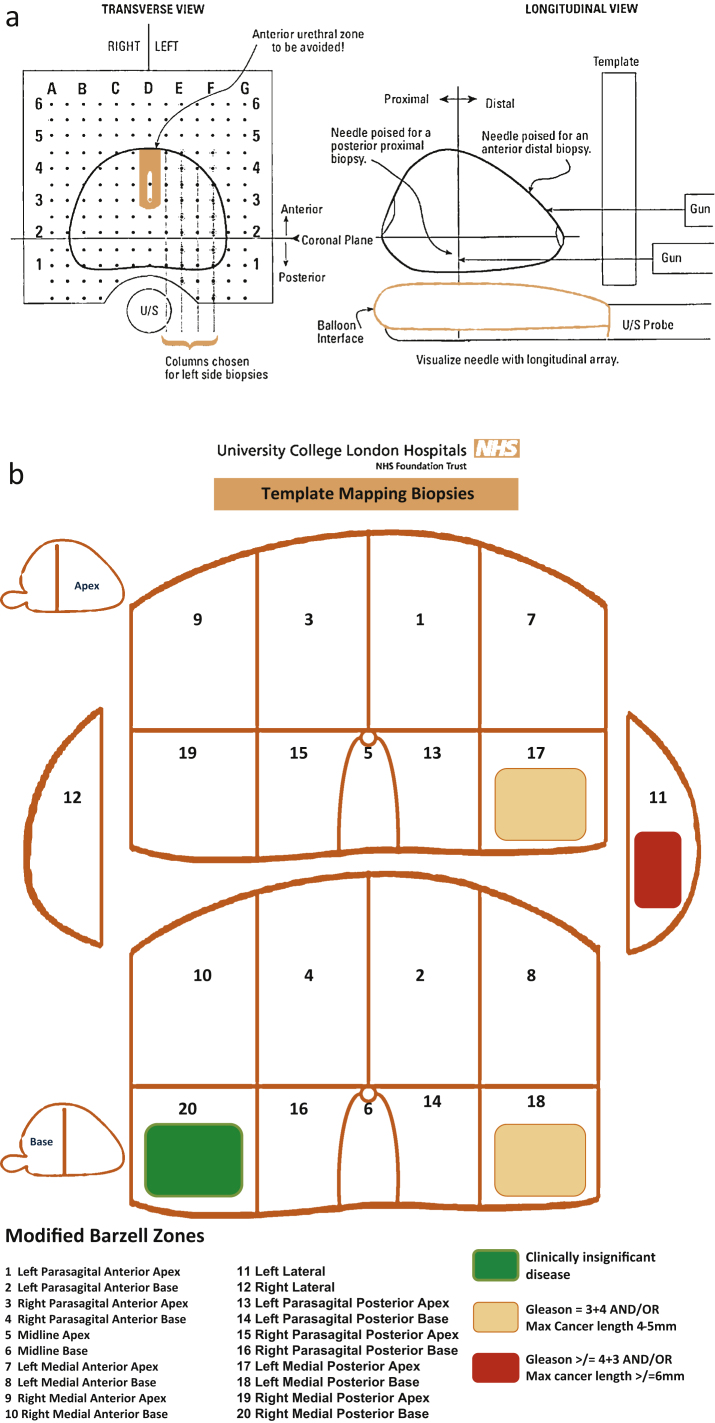
Template prostate-mapping biopsies. (a) Biopsies are taken every 5 mm through a template brachytherapy grid using a method described by Winston Barzell. Biopsies are still taken every 5 mm throughout the prostate, and two biopsies are taken from the same grid coordinate if the prostate is longer than the length of one core biopsy [19]. (b) Regional method used on template-mapping biopsy. Although 5-mm sampling is carried out, the biopsies are batched into 20 zones to limit pathology burdens. The colour coding of individual lesions/zones is based on Kirkham et al. [19]. In this case, index lesion ablation could be targeted to the left peripheral zone lesion and the low-volume, low-grade cancer in zone 20 left untreated. Reprinted from [18] with permission from Elsevier.

This study received exemption from ethics committee approval from the University College London Hospitals Joint Research Office. Our institutional TTPM biopsy registry includes all cases having this procedure. The majority of these patients were tertiary referrals to our institution with previous transrectal ultrasound–guided biopsies. TTPM biopsies were conducted using a method previously described, with cores taken every 5 mm throughout the prostate using a template grid (Fig. 1) [18]. Antibiotic prophylaxis was used with single-dose cefuroxime, gentamicin, and metronidazole at the time of induction. The complications were assessed on immediate postoperative findings and any hospital readmissions and were enquired of the patient at the 4–6-wk follow-up visit. The cancer risk group was determined using the US National Comprehensive Cancer Network (NCCN) guidelines. Locoregional radiologic staging was performed using prostate magnetic resonance imaging (MRI), and distant metastases were ruled out using a pelvic MRI and radioisotope bone scan in any man with a Gleason score ≥7 on any histology, prostate-specific antigen ≥10 ng/ml, or clinical/MRI T stage ≥T3a. The T stage was based on MRI characteristic only and not on histology [19].

**Fig. 2:**
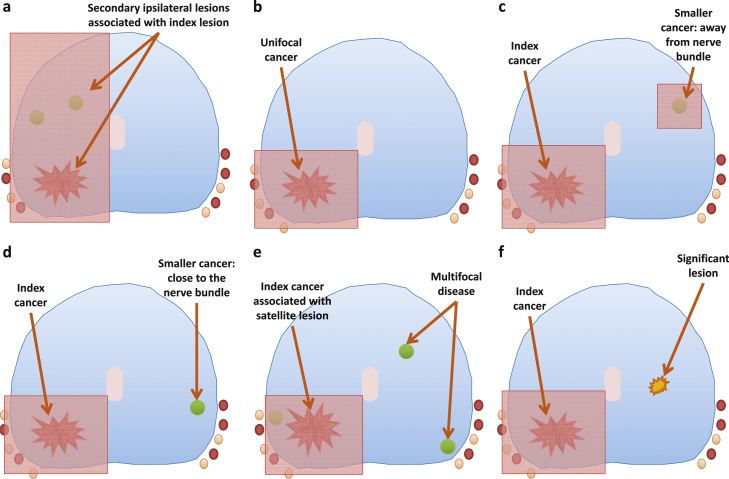
The morphologic characteristics of localised prostate cancers that were deemed suitable and not suitable for focal therapy: (a) unilateral disease, hemiablation; (b) unifocal disease, unifocal ablation; (c) bilateral bifocal disease, bifocal ablation; (d and e) index lesion with low-volume, low-grade lesion or lesions in contralateral areas, index lesion ablation; (f) bilateral high-volume or high-grade disease, not suitable for focal therapy.

Toxicity data were collected retrospectively through review of clinic notes and are reported for completeness, although they may be subject to recall bias. Criteria used to decide suitability for focal therapy were those used in prospective ethics committee–approved trials actively recruiting during the period of this study, with pathologic tumour features characterised according to a combination of cancer core length and Gleason grade [20] (Fig. 2). We have reported the results of two of these studies [9,11]. A third trial treating the index lesion is currently closed for analysis [18]. Our current multicentre focal therapy trial incorporates all these focal therapy strategies and will aim to recruit 150 men [20].

In summary, suitability for focal therapy required the cancer to be (1) unifocal, (2) unilateral, (3) bilateral/bifocal with at least one neurovascular bundle avoided, or (4) bilateral/multifocal with one dominant index lesion and secondary lesions with Gleason ≤3 + 3 and cancer core involvement ≤3 mm. The avoidance of the neurovascular bundle was based on ensuring that the posterior left or right quadrant of prostate tissue was not ablated. We accept that the neurovascular bundle is not a discrete bundle but has a more complex diffuse anatomic distribution. We felt that the avoidance of a posterior quadrant at least would avoid most of the ipsilateral nerves in question.

Because of the nonparametric nature of the data, a chi-square test or Spearman rank order for correlation was used, depending on expected values in the two-by-two tables. Cancer risk groups, in addition, were dichotomised at the low/intermediate and intermediate/high thresholds to reflect two schools of thought about the placement of focal therapy. First, some practitioners believe that focal therapy is an alternative for only those men suitable for active surveillance. Second, others have argued that focal therapy is an alternative for men with clinically significant cancer as a strategy that might overcome the harms of treatment but retain the cancer control benefits. A binary logistic regression model was also used, since the predictor variables were a combination of continuous and categorical variables and not normally distributed. Each logistic regression model used nine predictor variables. All tests were two-tailed and performed within SPSS statistical software v.17.0 (2010; IBM Corp., Armonk, NY, USA), and significance was defined as a *p* value <0.05.

## 3 Results

**Fig. 3:**
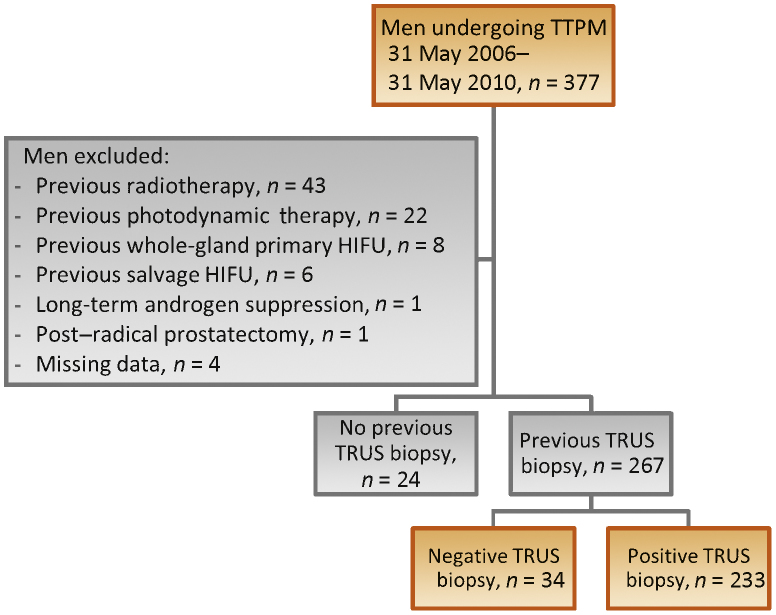
Flowchart demonstrating patient population characteristics. TTPM = transperineal template prostate mapping; TRUS = transrectal ultrasound; HIFU = high-intensity focussed ultrasound.

An unselected cohort of 377 men referred to our institution underwent TTPM biopsy between 2006 and 2010; of these men, 291 had no previous treatment and formed our cohort for analysis (Fig. 3, Tables 1 and 2). The side-effects of TTPM included perineal ecchymosis in 100% of the men (291 of 291); mild, self-resolving haematuria in most; haematuria requiring admission in 2% (6 of 291); urinary retention in 7% (20 of 291); urinary tract infection in 1% (3 of 291); scrotal skin cellulitis in 0.3% (1 of 291); and no sepsis. We did not routinely collate data on erectile dysfunction at baseline or follow-up, so the actual number with haematospermia is unknown.

Ninety-two percent of men with cancer (220 of 239 men) on TTPM biopsy were suitable for at least one form of focal therapy: hemiablation (22%, 53 of 239 men), unifocal ablation (31%, (73 of 239 men), bilateral/bifocal ablation (14%, 33 of 239 men), and index lesion ablation (26%, 61 of 239 men) (Table 3). Based on univariate analysis, being in the NCCN high-risk group was a statistically significant predictive factor for men not suitable for focal therapy, although numbers were small (Table 4). When dichotomising between low- and intermediate/high-risk groups, the proportion of men suitable for focal therapy decreased from 99% (84 of 85 men) to 91% (94 of 106 men), respectively (*p* = 0.005). When dichotomising between low/intermediate-risk compared with high-risk groups, 95% (166 of 175 men) compared with 75% (12 of 16 men) were suitable for focal therapy (*p* = 0.002).

On binary logistic regression modelling that incorporated transrectal biopsy parameters, we found no statistically significant predictive factor for focal therapy suitability. However, when TTPM biopsy variables were used instead, stage (specifically, radiologic T2c) was a significant negative predictor (*p* = 0.001) (odds ratio: 0.001 [95% confidence interval, 0.000–0.048]) (Table 5).

## 4 Discussion

Approximately 90% of men presenting with low- and intermediate-risk disease in our cohort were suitable for at least one focal therapeutic strategy using TTPM biopsy as a means to localise individual PCa lesions.

Our study has a number of limitations. First, as a tertiary centre, we had men presenting to us who were interested in focal therapy. This situation might have led to selection bias, as men with larger cancer burdens on transrectal biopsy may not have sought further risk stratification or trials in focal therapy. This bias is difficult to quantify. Second, as there is no clear consensus as to which risk category for focal therapy should be investigated [3–6,15,16], our inclusion of intermediate- and high-risk groups may be controversial. We have tried to reflect this lack of consensus by describing all risk groups in an open manner. Third, although we found that clinical T stage was the only negative predictor for suitability of focal therapy, it must be noted that clinical T stage does not correlate very well with final pathologic stage or final oncology outcome after definitive treatment. Fourth, it is clearly important to remember that while defining the patient population is important and facilitates decision making in clinical practice and research, focal therapy has no long-term outcomes on disease control and is thus not yet considered standard care. Finally, there is no gold standard control with which to compare the results of TTPM biopsy; hence, the accuracy of TTPM biopsy in tumour localisation may be questioned. However, both simulation models [21] and a radical prostatectomy comparison study [22] reflect a high level of fidelity. At the same time, we acknowledge that lack of definitive final histology could have an unquantifiable bias in the current study.

A large study population, accurate data collection, and mapping of individual cores of the TTPM biopsies for every patient added strength to the study. The different focal therapy strategies are based on our prospective trials and are thus not just theoretical concepts. We have previously shown that of men with low- and intermediate-risk disease who have undergone radical prostatectomy, between 51% and 68% would have been suitable for a form of focal therapy including index lesion ablation [23,24]. Other researchers have identified that only one-fifth to one-third of men may be suitable [25]. These differences may be due to controversy surrounding the concept of the index lesion and whether it is safe to leave low-grade, low-volume lesions untreated. We have included this concept as a focal therapeutic strategy, since men are currently being treated in this manner within the context of ethics committee–approved trials [17–19]. Indeed, many focal therapy series in which transrectal biopsy is used to localise lesions are likely to be treating by an index lesion ablation de facto.

Our study has relevance on a number of levels. First, when patients wish to explore focal therapy and are recommended to have a general anaesthetic and multiple biopsies, which carry some additional toxicity, they are likely to want to know the odds that they might be found to have suitable disease for focal therapy. Second, physicians offering template biopsies with a view to focal therapy are better placed to advise and counsel while also being able to make a judgement on whether the additional resources are worthwhile for their particular health care setting. Third, with designs for randomised controlled trials of focal therapy compared with radical therapy being considered, there is a key issue about when to apply a template biopsy with respect to the timing of randomisation. If template biopsies are conducted prior to randomisation, men potentially go through a morbid, high-burden test that will have little clinical relevance if they are randomised to the control arm. If templates are conducted after randomisation and only in the focal arm, but a large proportion of men are then not suitable for focal therapy (therefore, they have radical therapy), this situation would be problematic from an intention-to-treat analysis. Our study has shown that template biopsies after randomisation would not necessarily lead to significant rates of whole-gland therapy in the focal therapy arm.

There are no widely accepted standards for disease localisation in focal therapy, since studies have shown that transrectal biopsy on its own is not sufficient [26]. However, TTPM biopsy is more invasive and requires considerable health care resources. Its major advantage is high sensitivity and negative predictive value for detecting and ruling out lesions with 0.5-ml volume [28]. Since our early focal therapy trials formed some of the first trials and followed a phased programme [27], we decided to use TTPM biopsy to ensure, with a high degree of confidence, that clinically significant disease was not left untreated. Since then, evidence on multiparametric MRI shows that this modality might have negative predictive values of 90–95% for ruling out clinically significant PCa (Gleason ≥3 + 4 and/or lesion ≥0.5 ml) using whole-mount prostatectomy [28,29] or TTPM [30] as a reference standard and thus might have a role in focal therapy disease localisation.

## 5 Conclusions

The success of tissue-preserving focal therapy is dependent on appropriate patient selection. This selection necessitates an accurate investigative tool that can exclude significant cancer outside the area intended to be ablated while precisely localising individual cancer lesions, which are to be selectively destroyed. When such a test, TTPM biopsy, was applied to men with low- and intermediate-risk PCa, most men were found to be suitable for a tissue preservation strategy. Whether such a tissue-preserving strategy gives long-term favourable oncologic outcomes is currently being evaluated by various ongoing focal therapy trials.

  ***Author contributions:*** Hashim U. Ahmed had full access to all the data in the study and takes responsibility for the integrity of the data and the accuracy of the data analysis.  

*Study concept and design:* Ahmed, Freeman, Emberton.

*Acquisition of data:* Ahmed, Singh, Dalton, Stevens, Arya, Freeman, Jameson, Barbouti, Gurung, Anele.

*Analysis and interpretation of data:* Ahmed, Singh, Arya.

*Drafting of the manuscript:* Singh, Ahmed.

*Critical revision of the manuscript for important intellectual content:* Emberton, Ahmed, Singh, Stevens, Arya.

*Statistical analysis:* Ahmed.

*Obtaining funding:* Ahmed, Emberton.

*Administrative, technical, or material support:* Freeman, Jameson.

*Supervision:* Ahmed.

*Other* (specify): None.  

***Financial disclosures:*** Hashim U. Ahmed certifies that all conflicts of interest, including specific financial interests and relationships and affiliations relevant to the subject matter or materials discussed in the manuscript (eg, employment/affiliation, grants or funding, consultancies, honoraria, stock ownership or options, expert testimony, royalties, or patents filed, received, or pending), are the following: Mark Emberton and Hashim U. Ahmed received funding from Sonacare Inc. for an investigator-led focal therapy trial using the Sonablate 500 HIFU device; received free use of the Nanoknife device from Angiodynamics for an investigator-led clinical trial of focal therapy; and received funding from the Medical Research Council (UK), Pelican Cancer Foundation, St Peters Trust, Prostate Cancer UK, Wellcome Trust, NIHR-i4i, and NIHR-HTA. Mark Emberton received consultancy payments from Sonacare, GSK, and Steba Biotech and has share options and is a director in Nuada Medical Ltd. Alex Freeman has share options in Nuada Medical Ltd. Manit Arya received funding from Orchid (a male cancer charity), Barts, and The London Charity.  

***Funding/Support and role of the sponsor:*** This study was funded by an MRC fellowship grant awarded to HUA and supported by the National Institute for Health Research University College London Hospitals Biomedical Research Centre.

## Figures and Tables

**Table 1:** Baseline characteristics in 291 men undergoing transperineal template prostate-mapping biopsy

Baseline characteristics	Value
Age, yr, median (IQR) (overall range)	61 (9) (40–81)
Serum PSA, ng/ml, median (IQR) (overall range)	6.8 (5.5) (2.1–24.8)
Prostate volume, ml, median (IQR) (overall range)	35.0 (18) (15–113)
PSA density, ng/ml per cubic centimetre, median (IQR) (overall range)	0.17 (0.14) (0.02–0.99)
Initial biopsy strategy, no. (%)	
TRUS biopsy	267 of 291 (92)
TTPM biopsy	24 of 291 (8)
Gleason (if positive on TRUS-guided biopsy), no. (%)	
6	
3 + 3	163 of 233 (70)
7	56 of 233 (24)
3 + 4	46 of 233 (20)
4 + 3	10 of 233 (4)
Missing	17 of 233 (6)
TRUS-guided biopsies	
Total cores, no., median (IQR) (overall range)	10 (4) (3–18)
Total positive cores, no., median (IQR) (overall range)	2 (2) (1–10)
Positive cores, %, median (IQR) (overall range)	6.0 (6.5) (1.2–24.0)
MCL, mm, median (IQR) (overall range)	3 (4) (1–14)
% MCL, median (IQR) (overall range)	25 (30) (1–100)
TRUS biopsy laterality, no. (%)	
Unilateral	199 of 233 (85)
Bilateral	23 of 233 (10)
Missing	11 of 233 (5)
Radiologic (MRI) stage, no. (%)	
T1c	85 of 239 (36)
T2a	105 of 239 (44)
T2b	27 of 239 (11)
T2c	5 of 239 (2)
T3a	17 of 239 (7)
Risk group (NCCN) after TRUS biopsy, no. (%)	
Low	102 of 233 (44)
Intermediate	98 of 233 (42)
High	16 of 233 (7)
Missing	17 of 233 (7)

IQR = interquartile range; PSA = prostate-specific antigen; TRUS = transrectal ultrasound; TTPM = transperineal template prostate mapping; MCL = maximum cancer length; MRI = magnetic resonance imaging; NCCN = National Comprehensive Cancer Network. Note: Of men with positive TRUS biopsy, 25 (12%) had a negative TTPM biopsy.

**Table 2:** Details of transperineal template prostate-mapping biopsies in 291 men

Characteristics	Value
Reason for undergoing TTPM biopsies, no. (%)	
Positive TRUS biopsy	233 of 291 (80)
Risk stratification	69 of 291 (24)
Focal therapy	164 of 291 (56)
Negative TRUS biopsy, persistent risk	34 of 291 (12)
Diagnostic (no previous TRUS biopsy)	24 of 291 (18)
TTPM biopsies	
Total cores, no., median (IQR) (overall range)	29 (18) (10– 0)
Core density (biopsies per cubic centimetre), median (IQR) (overall range)	1.1 (1.2) (0.4–7.5)
Total positive cores, no., median (IQR) (overall range)	8 (5) (2–31)
Positive cores, %, median (IQR) (overall range)	5.2 (6.8) (0.6–74.0)
MCL, mm, median (IQR) (overall range)	6 (5) (1–15)
% MCL, median (IQR) (overall range)	50 (55) (3–100)
Gleason (TTPM biopsies), no. (%)	
No cancer	52 of 291 (18)
3 + 3	96 of 291 (33)
Score 7	127 of 291 (44)
3 + 4	119 of 291 (41)
4 + 3	8 of 291 (3)
4 + 4	1 of 291 (0.3)
Not gradable	15 of 291 (5)
Risk group (NCCN) after TTPM, no. (%)	
Low	70 of 239 (29)
Intermediate	144 of 239 (60)
High	20 of 239 (8)
Missing	5 of 239 (2)
TTPM laterality, no. (%)	
Unilateral	94 of 239 (39)
Right	45 of 239 (19)
Left	49 of 239 (21)
Bilateral	145 of 239 (61)

TTPM = transperineal template prostate mapping; TRUS = transrectal ultrasound; IQR = interquartile range; MCL = maximum cancer length; NCCN = National Comprehensive Cancer Network. Note: Of men with positive TRUS biopsy, 25 (12%) had a negative TTPM biopsy.

**Table 3:** The proportion of men suitable for focal therapy following positive transperineal template prostate-mapping biopsy

Focal strategy	Value, no. (%)
Suitable for focal therapy	220 of 239 (92)
Not suitable for focal therapy	19 of 239 (8)
Unilateral disease (Fig. 2a and 2b)	
Suitable for focal therapy	126 of 239 (53)
Hemiablation (Fig. 2a)	53 of 239 (22)
Unifocal ablation (Fig. 2b)	73 of 239 (31)
Not suitable for focal therapy	0 (0)
Bilateral disease (Fig. 2c– 2f)	
Suitable for focal therapy	94 of 239 (39)
Bilateral focal ablation (Fig. 2c)	33 of 239 (14)
Index lesion ablation only (Fig. 2d–2f)	61 of 239 (26)
Not suitable for focal therapy	19 of 239 (8)

**Table 4:** The relationship of suitability for focal therapy and risk groups following transperineal template prostate-mapping biopsies

NCCN category based on TTPM biopsy	Unsuitable for focal therapy, no. (%)	Suitable for focal therapy, no. (%)	
Low	3 of 70 (4)	67 of 70 (96)	Spearman rank order correlation (expected cell frequency <5), *p* = 0.017
Intermediate	10 of 140 (7)	130 of 140 (93)	
High	5 of 18 (28)	13 of 18 (72)	
Low	3 of 70 (4)	67 of 70 (96)	Pearson chi-square, *p* = 0.179
Intermediate and high	15 of 158 (10)	143 of 158 (91)	
Low and intermediate	13 of 210 (6)	197 of 210 (94)	Spearman rank order correlation (expected cell frequency <5), *p* = 0.001
High	5 of 18 (28)	13 of 18 (72)	

TTPM = transperineal template prostate mapping; NCCN = National Comprehensive Cancer Network.

**Table 5:** The role of transrectal biopsy and transperineal template prostate-mapping biopsy parameters in combination with other clinical baseline parameters to predict subsequent suitability for focal therapy (binary logistic regression)

Variables	Odds ratio	*p* value
Variables for binary logistic regression model based on TRUS biopsy parameters		
Age	0.000	0.989
PSA	0.000	0.996
Total number of cores	0.000	0.990
Number of positive cores	0.000	0.972
Maximum cancer length	<0.001	0.989
Gleason score (with respect to Gleason 6)		
Gleason 7	<0.001	0.973
Volume	1.779	0.995
Stage (with respect to stage T1c)		1.000
Stage T2a	0.000	0.982
Stage T2b	0.000	0.987
Stage T2c	0.000	0.989
Stage T3a	0.000	0.991
NCCN risk (with respect to low risk)		1.000
Intermediate	0.000	0.979
High	<0.001	0.995
Variables for binary logistic regression model based on TTPM parameters		
Age	1.023	0.665
PSA	0.938	0.362
Volume	0.997	0.908
Stage (with respect to stage T1c)		0.007
Stage T2a	0.253	0.298
Stage T2b	0.041	0.084
Stage T2c	0.001	0.001
Stage T3a	0.000	1.000
NCCN risk (with respect to low risk)		0.835
Intermediate	2.306	0.548
High	<0.001	1.000
Total number of cores	1.019	0.475
Number of positive cores	0.937	0.254
Maximum cancer length	0.870	0.481
TTPM Gleason score (with respect to Gleason 6)		0.943
Gleason 7	1.472	0.733
Gleason 8	0.000	1.000

TRUS = transrectal ultrasound; TTPM = transperineal template prostate mapping; PSA = prostate-specific antigen; NCCN = National Comprehensive Cancer Network.
